# Oral Microbiome Resilience During SARS‐CoV‐2 Infection and Diversity Shifts After COVID‐19 Vaccination in a Hispanic Population

**DOI:** 10.1002/mbo3.70310

**Published:** 2026-05-06

**Authors:** Daniela Vargas‐Robles, Jean L. Santos Agrait, Jaleniz Suárez‐Pérez, Frances Vázquez, Anelisse Dominicci‐Maura, Carlos A. Sariol, Carmen Zorrilla, Josefina Romaguera, Filipa Godoy‐Vitorino

**Affiliations:** ^1^ Department of Microbiology and Immunology, University of Puerto Rico, School of Medicine Medical Sciences Campus San Juan Puerto Rico USA; ^2^ Department of Obstetrics and Gynecology University of Puerto Rico, School of Medicine, Medical Sciences Campus San Juan Puerto Rico USA; ^3^ Comprehensive Cancer Center, University of Puerto Rico Medical Sciences Campus San Juan Puerto Rico USA; ^4^ Unit of Comparative Medicine, Department of Medicine University of Puerto Rico‐Medical Sciences Campus San Juan Puerto Rico USA

**Keywords:** COVID‐19, hispanics, oral microbiota, vaccination

## Abstract

The relationship between SARS‐CoV‐2 infection and the oral microbiome remains poorly understood, particularly in the Hispanic population. Oral samples from 62 individuals (38 SARS‐CoV‐2 positive, 24 negative) were analyzed using 16S rRNA sequencing, comparing diversity and taxa by infection and symptoms. Longitudinal data from 11 participants assessed microbiome changes as the infection resolved over time. To assess the impact of vaccination, we further examined 68 consistently SARS‐CoV‐2–negative individuals with paired samples collected before and after vaccination. SARS‐CoV‐2 infection was not significantly associated with alpha diversity, while beta diversity showed a non‐significant but marginal trend (*p* = 0.051). *Prevotella nanceiensis* was consistently depleted in infected individuals, even after excluding recent antibiotic users, suggesting a reproducible association with infection status rather than a diagnostic marker. Among infected participants, mucosa‐related symptoms were associated with lower *Veillonella parvula* abundance. Longitudinal data revealed stable microbiome profiles with slightly reduced variance in alpha diversity following viral clearance. In contrast, COVID‐19 vaccination in consistently negative individuals was associated with significant increases in Shannon (*p* = 0.050) and Simpson diversity (*p* = 0.017), indicating greater evenness without expansion of richness. Beta diversity analyses showed vaccination‐related shifts in community composition (PERMANOVA *p* = 0.026), with increases in *Treponema*, *Campylobacter*, *Oribacterium*, and *Selenomonas*, and a decrease in *Haemophilus*. The oral microbiome of Hispanics with mild SARS‐CoV‐2 infection appeared resilient, with only subtle taxonomic alterations. In contrast, COVID‐19 vaccination was associated with short‐term increases in diversity and compositional shifts, highlighting its influence on oral microbial ecology.

## Graphical abstract

The oral microbiome in Hispanics remains largely resilient during mild SARS‐CoV‐2 infection, showing minimal diversity changes, while COVID‐19 vaccination induces increased microbial evenness and compositional shifts. These findings highlight limited infection impact but suggest vaccination transiently reshapes oral microbial ecology.

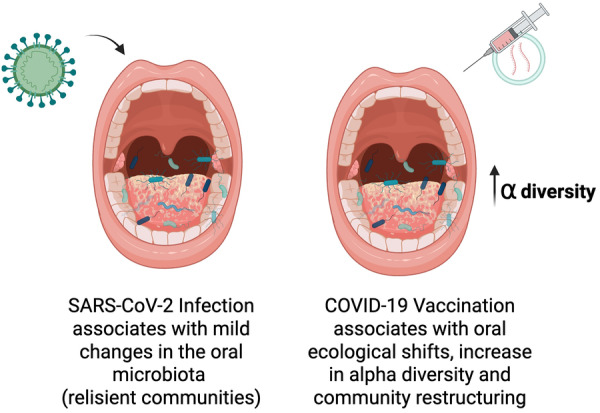

## 1 Introduction

The COVID‐19 pandemic caused by SARS‐CoV‐2 has had profound global health impacts. Although respiratory symptoms are the most common, COVID‐19 can affect multiple organ systems, including the oral cavity (reviewed by (Dziedzic and Wojtyczka 2021)). There has been substantial evidence in other studies that show that viruses and the microbiome engage in essential reciprocal interactions and that the microbiome may regulate and, in turn, viruses control it via distinct mechanisms (12). Dysbiosis or disease progression is favored by viruses that alter the microbiota, including periodontal inflammation in the oral cavity (Chen et al. 2018; Herrero et al. 2018; Soffritti et al. 2021; Ortiz et al. 2022; Deng et al. 2025). SARS‐CoV‐2 is known to enter the body mainly through the oropharynx, where it encounters epithelial cells expressing the ACE2 and TMPRSS2 viral receptors (Hoffmann et al. 2020). The virus has been found in saliva (Chopoorian et al. 2023); thus, existing oral microbiota may impact the capacity of SARS‐CoV‐2 to initiate or facilitate infections.

COVID‐19 negatively impacts oral health through direct viral effects on the salivary glands, taste/smell, oral mucosa, and microbiota balance. Intensified therapies and multi‐drug treatments can aggravate pre‐existing autoimmune oral conditions and cause side effects such as stomatitis, ulcers, and dry mouth, as well as disrupt the oral microbiota balance (Lovato et al. 2020; Sabino‐Silva et al. 2020). In severely ill hospitalized patients, oral health deteriorates due to neglected mouth care, mouth breathing, and hyposalivation (Wu et al. 2020). These factors, combined with an impaired immune system, increase the risk of opportunistic infections, such as fungal infections, dry mouth, ulcerations, and gingivitis. Several studies have reported oral microbiome alterations associated with SARS‐CoV‐2 infection, including reduced microbial diversity and shifts in community composition(Ganesan et al. 2024). These dysbiotic patterns appear to be more pronounced in severe cases of COVID‐19 (Armstrong et al. 2023).

Most of the investigations about human microbiome–COVID‐19 associations have been conducted in multiethnic or predominantly non‐Hispanic populations. Those U.S.‐based studies including Hispanics have not stratified the results by ethnicity (Miller et al. 2021). Given that baseline oral microbial profiles vary by race, ethnicity, and acculturation (Hoffman et al. 2018), with Hispanics exhibiting distinct bacterial signatures and higher diversity compared to other groups, and considering that environmental and dietary factors further shape the oral microbiome, it remains unclear whether the findings from U.S. or non‐Hispanic cohorts can be directly extrapolated to Hispanics living in their countries of origin (Gupta et al. 2017). This knowledge gap underscores the need to investigate oral microbiome–COVID‐19 associations in Hispanic populations outside the United States, where the effects of acculturation disappear (Table 1).

**Table 1:** Participant characteristics stratified by SARS‐CoV‐2 status (negative vs positive).

Variable	Overall	COVID‐19 status		*p* ^a^
		Negative	Positive	
*n*	62	38	24	
Age (mean (SD))	37.70 (11.98)	40.30 (13.18)	33.58 (8.50)	0.030*
Sex = Male (%)	17 (27.4)	9 (23.7)	8 (33.3)	0.591
BMI (mean (SD))	28.07 (5.35)	28.38 (5.77)	27.58 (4.70)	0.572
Physical Activity Sports = Yes, > 0 days week (%)	20 (32.3)	15 (39.5)	5 (20.8)	0.211
Antibiotics last 2months = Yes (%)	18 (30.0)	7 (19.4)^b^	11 (45.8)	0.058
Diabetes type 2 = Yes (%)	3 (4.8)	3 (7.9)	0 (0.0)	0.422
Nicotine cigarret curr = Yes (%)	3 (4.8)	2 (5.3)	1 (4.2)	1.000
Medicacal or recreatinal cannabis = Yes (%)	4 (6.5)	3 (7.9)	1 (4.2)	0.959
Hypertension = Yes (%)	12 (19.4)	8 (21.1)	4 (16.7)	0.924
Smoking curr vap cigar w and wo nicot = Yes (%)	4 (6.5)	2 (5.3)	2 (8.3)	1.000
Ever periodontal disease = Yes (%)	2 (3.2)	1 (2.6)	1 (4.2)	1.000
Rhinitis = Yes (%)	7 (11.3)	5 (13.2)	2 (8.3)	0.863
Bronchial asthma = Yes (%)	9 (14.5)	6 (15.8)	3 (12.5)	1.000
Ever cancer = Yes (%)	2 (3.2)	2 (5.3)	0 (0.0)	0.686
COVID19 vaccinaed ever adj = Yes (%)	7 (11.9)	1 (2.7)^c^	6 (27.3)^b^	0.016*
Past COVID‐19 (suspected or confirmed) = Yes (%)	11 (17.7)	0 (0.0)	11 (45.8)	< 0.001*

^a^ *p*‐values were generated from *T*‐test or Kruskal‐Wallis for continous variables or Chi‐squared or Fisher exact test for categorical variables.

^b^ 2 missing values.

^c^ 1 missing value.

* *p*‐values < 0.05.

This study hypothesized an association between SARS‐CoV‐2 infection and oral microbiome composition in a Hispanic population from Puerto Rico. Differences in the diversity and abundance of oral microbial taxa are expected between SARS‐CoV‐2‐positive and negative individuals, along with correlations between microbiome profiles and COVID‐19 symptoms. We compared oral microbiome profiles between SARS‐CoV‐2 positive and negative individuals, examined correlations with COVID‐19 symptoms, and analyzed changes in individuals transitioning from positive to negative status as well as the influence of vaccination in the oral microbiome of SARS‐CoV‐2‐negative individuals.

## 2 Materials and Methods

### 2.1 Study Design and Ethical Approval

This observational study was conducted at the University Adult Hospital (UH) and the University of Puerto Rico Medical Sciences Campus (MSC) between July 20, 2020, and May 18, 2022. Ethical approval was obtained from the Institutional Review Board (IRB) of the University of Puerto Rico School of Medicine (protocol #B1760120), and authorization for the use of biological samples was granted by the Institutional Biosafety Committee (IBC protocol #94320). All study procedures adhered to established ethical standards and were conducted in accordance with the principles of informed consent.

### 2.2 Participant Recruitment and Eligibility

A total of 313 participants were recruited for the study, comprising healthcare workers, non‐healthcare workers, pregnant women, university students, and staff affiliated with the medical sciences campus. Recruitment was promoted through flyers and email announcements distributed across the university community. Eligible participants were 21 to 70 years old and met one or more of the following inclusion criteria: (1) employment at UDH or MSC, (2) close family members of healthcare personnel, or (3) pregnancy at the time of enrollment. Exclusion criteria included individuals younger than 21 or older than 70 years, those unable to provide informed consent due to cognitive or mental health conditions, and those who did not meet the study criteria. Upon arrival, participants provided written informed consent, completed a structured questionnaire, and submitted biological samples for SARS‐CoV‐2 testing. The questionnaire collected information on socio‐demographic characteristics, health history, vaccination status, hygiene practices, nutrition, lifestyle factors (e.g., smoking, alcohol consumption, physical activity), and COVID‐19‐related symptoms and exposures.

### 2.3 Sample Collection and Processing

Nasopharyngeal swabs were collected by trained healthcare professionals for SARS‐CoV‐2 PCR testing. In this project, additional self‐collected biological samples included saliva, oral, and rectal swabs, as well as whole blood. Samples were double bagged, stored on ice packs, and transported to the MSC Laboratory of Parasite, Immunology, and Pathology for processing.

PCR tests were done for the nasopharyngeal swabs as well as for DNA extracted from saliva samples using FDA approved molecular methods (RT PCR) using TaqPath™ COVID‐19 Combo Kit (Thermo Fisher Scientific) in accordance with CDC guidelines (CDC‐006‐00019, Revision: 02). The assay employed primers and probes targeting specific SARS‐CoV‐2 genomic regions, and fluorescence signals were monitored during each amplification cycle to detect viral presence. Each run included a positive control consisting of RNA from the Human coronavirus 229E strain (ATCC VR‐740D). All reactions were performed following CDC‐recommended protocols to ensure high specificity and sensitivity of viral detection. Results from PCR tests were communicated within 24–48 h, and participants testing positive were contacted for confirmatory testing and followed weekly until a negative result was obtained.

Saliva‐derived genomic DNA was extracted using the QIAGEN DNeasy PowerSoil Pro Kit (250) and automated on a Qiagen QIAcube robot. DNA quantification was performed with a Qubit® 2.0 Fluorometer (Thermo Fisher Scientific). The 16S rRNA V4 region was amplified using universal primers REF and sequenced on an Illumina MiSeq platform.

### 2.4 Sample Inclusion

From the biobank of oral samples collected during the COVID‐19 pandemic, we selected 120 non‐pregnant individuals aged 21–60 years. To examine the impact of SARS‐CoV‐2 infection, we included individuals who tested positive for SARS‐CoV‐2 as cases and those who tested negative as controls. For the negative control group, we excluded participants who reported any COVID‐19–related symptoms (difficulty breathing, diarrhea, fever > 38°C, loss of smell or taste, muscle pain, headaches, sore throat, chest pain or tightness, nasal congestion, sputum/phlegm, or rash) or a history of suspected COVID‐19 infection, to minimize the risk of including undetected cases. We included the COVID negative individuals as subset of individuals from our larger cohort, who tested negative for SARS‐CoV‐2 by PCR and reported no history of suspected or confirmed COVID‐19 infection. These participants, classified under the variable *COVID_consensus_COVID19*, were confirmed as COVID‐negative based on both molecular testing and self‐reported clinical history. To determine whether COVID‐19 vaccination influenced the oral microbiome, we analyzed a subset of confirmed SARS‐CoV‐2–negative individuals who reported never having had a suspected or confirmed infection.

### 2.5 Sequence Processing

ASVs were generated using DADA2 (Callahan et al. 2016) algorithm implemented in QIIME2(Bolyen et al. 2019), and taxonomic classification was performed using the q2‐feature‐classifier plugin (Bokulich et al. 2018) with GreenGenes and NCBI data (Usyk et al. 2020). The resulting feature table and metadata were imported into R v.4.4.0 (Team, R. C. 2024) using the ***qiime2R*** (Bisanz 2018) R function for downstream analysis. We removed spurious ASVs (< 10seqs or present in only one sample) and those ASVs that were unclassified to at least the order level. A total of 29 highly abundant and unclassified species (with more than 10,000 sequences across the entire dataset) were manually blasted on the NCBI. The species identity chosen was that with 100% identity and the highest total score. If multiple accessions had the same values, the accession with both genus and species fully identified was selected. We applied rarefaction and verified with Good's coverage index using *the QsRutils*(Quensen 2020) R package to ensure that the chosen limit captured sample diversity. Representative rarefaction achieved a median Good's index of 90% and a mean of 99.3% at 3,450 sequences/sample, losing nine SARS‐CoV‐2‐negative samples. Microbiome diversity metrics were computed using the *vegan*(Oksanen et al. 2007) and all data analysis and visualization were conducted using the *phyloseq* (McMurdie and Holmes 2013) and *tidyverse* (Wickham et al. 2019) packages.

The raw sequences and their associated metadata have been deposited in ENA study number ERP18563 with accession number PRJEB104336. The complete code used for the bioinformatics and statistical analysis presented in this study is publicly available in the following GitHub repository: https://github.com/UPR-MicrobiomeLab/COVID-19-2025.

### 2.6 Statistical Analysis

We used two analytical approaches. First, we compared SARS‐CoV‐2‐positive versus ‐negative individuals. Second, we restricted the analysis to SARS‐CoV‐2‐positive samples only. In this subset, rather than using the conventional symptomatic versus asymptomatic classification employed in other studies, we separated individuals based on the presence of mucosa‐related symptoms (throat pain, nasal congestion, loss of smell, loss of taste, conjunctivitis, excessive tearing, dry cough, difficulty breathing, and sputum/phlegm). This decision was motivated by the distribution of symptoms in our cohort; only six individuals reported neither mucosa‐related nor systemic symptoms (e.g., fatigue, diarrhea, high fever, muscle pain, headache, vomiting, and joint pain). Defining groups in this manner resulted in a more balanced comparison (12 with and 12 without mucosa‐related symptoms) and was better aligned with our focus on the oral microbiome.

We also addressed whether COVID‐19 vaccination influenced the oral microbiome; therefore, we analyzed a subset of confirmed SARS‐CoV‐2‐negative individuals who reported never having had a suspected or confirmed infection. In this group, paired samples collected before and after vaccination (at 3 and/or 6 months) were compared to each other. Because all participants remained consistently SARS‐CoV‐2 negative, no exclusion based on COVID‐19‐related symptoms were applied.

To compare demographic and clinical variables among groups, t‐tests or Kruskal‐Wallis tests were used for numerical data, while Fisher's exact test was applied to categorical data.

Microbial diversity analyses included beta diversity, alpha diversity, and discriminant taxon assessments. Beta diversity was evaluated using an Aitchison distance matrix computed from the filtered but non‐rarefied ASV tables, and comparison between groups was assessed using Permutational Multivariate Analysis of Variance (PERMANOVA(Anderson 2014)) with the “adonis2” and PERMDISP test with “betadisper” (Oksanen et al. 2007) R functions. Alpha diversity metrics (observed and Shannon indices) were calculated using rarefied data to account for differences in sequencing depth and were evaluated using linear models. The identification of specific discriminant microbial taxa was assessed using MaAsLin2 (Mallick et al. 2021) (Multivariable Association models) with a previous filtering to include only taxa in ≥ 20% of samples and with ≥ 15% relative abundance. MaAslin2 was run across multiple taxonomic levels (ASV, species, genus, family, order, class, and phylum) adjusting by recent antibiotic use, age, and sex.

Prior to evaluating SARS‐CoV‐2 status as the primary exposure of interest, we conducted an exploratory screening of demographic and clinical variables to identify potential confounders associated with oral microbial alpha diversity. The screened variables included recent antibiotic use, chronic conditions (e.g., asthma, hypertension, and cancer), respiratory symptoms (e.g., rhinitis), physical activity, and COVID‐19 vaccination status. Recent antibiotic use, maternal age, sex, and BMI were included as covariates in the models, given their consistent associations with oral microbiome diversity reported in the literature. This was the full model:

Beta/Alpha/Taxaabundance~SARS−CoV−2status+Antibiotics_last_2months+BMI+Sex+Age.

The optimal models were selected automatically using the “step” R function for alpha diversity and manually for beta diversity, where we removed BMI since it was found to be irrelevant. To retain all microbiome data, missing information on one individual's age was replaced with the median age of the studied population. However, missing data from two individuals regarding antibiotic use were not imputed. Given that 20 individuals had either used antibiotics in the preceding 2 months or did not respond to the inquiry, additional analyses were conducted, excluding these individuals.

### 2.7 Temporal Analysis

Longitudinal changes in the oral microbiome were assessed in 11 participants with paired samples collected during the SARS‐CoV‐2‐positive and ‐negative phases (mean interval = 184 days, range 106–216 days). Alpha diversity (Shannon index, observed richness) was compared using paired Wilcoxon signed‐rank tests, with correlations to interval length assessed using Pearson's correlation. Temporal variance differences in alpha diversity were tested using the Pitman‐Morgan test, and directional changes (increase vs. decrease in alpha diversity) were examined using Fisher's exact test. Beta diversity (Aitchison distance) was analyzed using PERMANOVA, restricting permutations within individuals (Patient_ID) to account for repeated measurements.

### 2.8 Temporal Analysis: Influence of Vaccination in Negative SARS‐CoV‐2 Patients

To evaluate the influence of COVID‐19 vaccination, we restricted the analysis to non‐pregnant individuals who were consistently SARS‐CoV‐2 negative (never suspected and confirmed infection during the sampling). For each participant, paired samples were selected as follows: the first visit was used as the *Before* sample, and the second or third visit as the *After* sample. Vaccination status at the *After* visit was used to classify individuals into two groups (PostVaccinated = Yes/No), which was propagated to the paired *Before* samples.

For alpha diversity, we used linear mixed‐effects models (LMMs) fitted separately for each alpha diversity metric using “lmerTest” (Kuznetsova et al. 2017) R function. The models included fixed effects for Period (*Before/After*), Vaccination (Yes/No), period * vaccination interaction, age, BMI, sex, and recent antibiotic use, with a random intercept for participant ID to account for repeated measures.

The estimated marginal means (EMMEANS) and pairwise contrasts were obtained using the emmeans package (Lenth 2023).

Beta diversity was assessed using the Aitchison distance. Paired PERMANOVA analyses were performed with adonis2, stratifying permutations by participant ID to account for within‐subject repeated measures and adjusting for age, BMI, sex, and recent antibiotic use. Ordination was subsequently carried out via non‐metric multidimensional scaling (NMDS).

Beta/Alpha/Taxaabundance~Period∗Vaccination+Age+BMI+Sex+Antibiotics_last_2months+(1|Patient_ID).

### 2.9 AI Tools

AI‐assisted tools (ChatGPT 5‐Auto and Papperpal) were used to format tables, write R code, and improve the quality of the written manuscript. All scientific content was verified by the authors.

## 3 Results

### 3.1 Population Study Characteristics

A total of 120 participants, contributing 189 oral samples, were included in this study. To assess the impact of SARS‐CoV‐2 infection on the oral microbiome, we analyzed a subset of 62 individuals (24 SARS‐CoV‐2‐positive and 38 SARS‐CoV‐2‐negative). Within this subset, a longitudinal subgroup of 11 participants (22 paired samples) was evaluated to examine temporal changes in the oral microbiome within individuals. Finally, to evaluate the influence of vaccination among ever‐infected participants, an additional 58 SARS‐CoV‐2‐negative individuals (116 samples) were analyzed, comprising 43 unvaccinated individuals and 15 who had received at least one COVID‐19 vaccine dose.

Among the first subset (*n* = 62), 45.8% of SARS‐CoV‐2‐positive individuals reported a prior history of suspected or confirmed COVID‐19, whereas none of the SARS‐CoV‐2‐negative individuals reported previous symptoms or positive diagnosis. None of the confirmed SARS‐CoV‐2 negative cases were suspected to have had COVID‐19 or related symptoms. Vaccination was reported by seven of these participants (11.9%), six of whom belonged to the SARS‐CoV‐2 positive group (*p* = 0.016). Vaccinations were administered in all cases prior to the onset of SARS‐CoV‐2 infection. Among them, five received the Pfizer‐BioNTech vaccine, and two were unsure of the brand of their vaccine. The study population had an average BMI in the overweight range and was predominantly female, comprising 45 women and 17 men; both variables were different between groups (*p* > 0.050). SARS‐CoV‐2‐positive individuals were significantly younger (*p* = 0.028). Additionally, 30% of the participants reported antibiotic use in the past 2 months, which showed a marginal but not statistically significant difference between the groups (*p* = 0.058). Other factors, such as comorbidities, clinical history, and lifestyle, showed no significant intergroup differences (*p* > 0.050). No participants reported HIV, type 1 diabetes, Crohn's disease, or Hepatitis B or C.

### 3.2 COVID‐19 Symptoms Characterization Among SARS‐CoV‐2 Infected Patients

**Figure 1:**
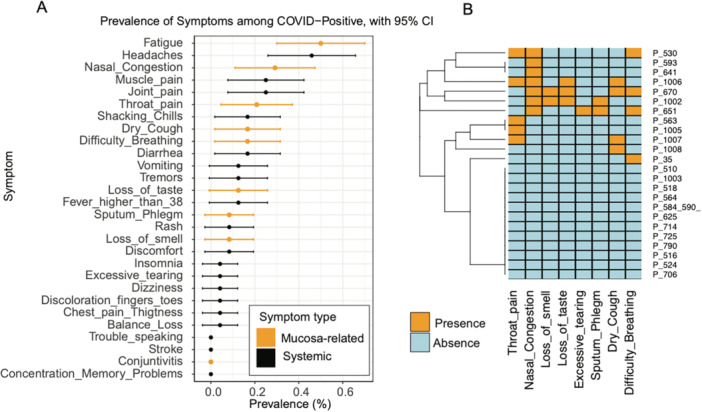
Prevalence and clustering of COVID‐related symptoms among SARS‐CoV‐2 infected patients. (A) Prevalence of self‐reported symptoms among COVID‐19 positive individuals. This dot plot shows the proportion of participants who reported each symptom. Error bars represent the 95% confidence intervals. (B) Exclusively mucosa‐related symptoms co‐occurrence and hierarchical clustering. A heatmap displaying the co‐occurrence of selected symptoms across individuals, with hierarchical clustering indicating the symptom groupings.

Among the 24 SARS‐CoV‐2‐positive individuals, only two were hospitalized during the infection, although none were intubated or ventilated. No patient reported stroke, conjunctivitis, difficulty speaking, concentration or memory problems, or loss of balance. The most prevalent symptoms were fatigue (50.0%), headache (45.8%), and nasal congestion (29.2%) (Figure 1A). A total of 50.0% of the participants (12/24) exhibited at least one mucosal symptom, with nasal congestion and throat pain being the most frequently reported symptoms (Figure 1B).

### 3.3 SARS‐CoV‐2 Infection and Oral Microbiome Association (Alpha and Beta Diversity)

**Figure 2:**
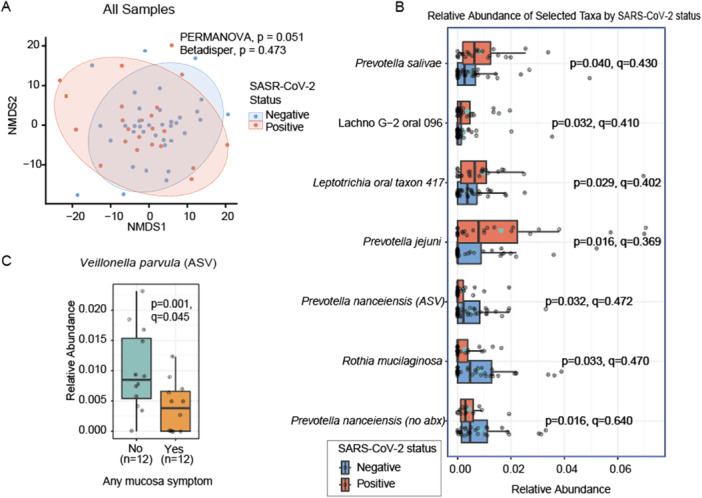
Oral microbiome profiles according to SARS‐CoV‐2 status and mucosal symptoms. (A) Non‐metric multidimensional scaling (NMDS) based on Aitchison dissimilarity, with samples colored by SARS‐CoV‐2 status and ellipses representing 95% confidence intervals. (B) Boxplots showing the relative abundance of selected oral taxa according to SARS‐CoV‐2 infection status, including species‐ and ASV‐level features. (C) Boxplot of the relative abundance of *Veillonella parvula* (ASV) stratified by the presence or absence of mucosal symptoms. Boxplots show the medians and interquartile ranges, with individual sample points and green stars indicating the group means. P‐ and q‐values from MaAsLin2 models are displayed for each comparison; only taxa with nominal statistical significance are shown.

We found no significant association between SARS‐CoV‐2 infection status and oral microbiome diversity, in alpha (Supporting Information S2: Figure S1) and marginal in beta diversity analyses (*p* = 0.051; *R*² = 2.3%; Figure 2). No significant differences in dispersion were observed between SARS‐CoV‐2‐positive and ‐negative individuals (*p* = 0.473, Supporting Information S1: Table S1). In contrast, recent antibiotic use (within the past 2 months) consistently predicted lower oral microbial richness and diversity (alpha: richness *p* = 0.013, diversity *p* = 0.016; beta: *p* = 0.030, *R*² = 2.5%; Supporting Information S1: Tables S1–S2). Age was negatively associated with diversity (*p* = 0.042) but not with richness (*p* = 0.110, Supporting Information S1: Table S2). Age was included as a covariate in all MaAsLin2, alpha diversity, and beta diversity models to account for its potential influence on microbial composition. Notably the SARS‐CoV‐2‐positive group was younger than the negative group, therefore residual confounding by age cannot be excluded. Therefore, these results should be interpreted with care.

Given the consistent effect of antibiotic use, we conducted a secondary analysis excluding individuals with a recent antibiotic exposure history. In this subset, SARS‐CoV‐2 status remained non‐significant for alpha diversity (Observed ASV: *p* = 0.126; Shannon not retained in the model, Supporting Information S2: Figure S1) and reduced significance for beta diversity (*p* = 0.278, Supporting Information S2:Figure S1). No differences in dispersion were detected (*p* = 0.779, Supporting Information S1: Table S1).

We also examined the impact of mucosa‐related symptoms in SARS‐CoV‐2‐positive individuals (*n* = 24). These symptoms were not significantly associated with alpha diversity (*p*‐values not retained in the model, Supporting Information S2: Figure S1), beta diversity (*p* = 0.471, Figure S1), or dispersion (*p* = 0.384, Supporting Information S2: Figure S1), even if we excluded recent antibiotic used (*p* = 0.379, Supporting Information S2: Figure S1).

### 3.4 MAasLin2 Analysis

SARS‐CoV‐2‐positive individuals showed higher relative abundance of several oral taxa with nominal significance (*p* < 0.050; *q* > 0.250), including *Prevotella jejuni, Prevotella salivae, Leptotrichia* sp. (oral taxon 417), and Lachnospiraceae [G‐2] (oral taxon 096). In contrast, *Rothia mucilaginosa* and *Prevotella nanceiensis* were depleted. These associations were consistent across species, genera, and ASV taxonomic levels (Figure 2). All models were adjusted for recent antibiotic use, age, and sex. *Leptotrichia* sp. and Lachnospiraceae [G‐2] (as it appears in GreenGenes2 database) were also significantly reduced in individuals recently exposed to antibiotics (Supporting Information S1: Table S3), suggesting their association with SARS‐CoV‐2 might reflect shared microbial responses rather than a virus‐specific effect. To test robustness, we repeated the analysis after excluding individuals with recent antibiotic use. In this antibiotic‐free subset, *Prevotella nanceiensis* remained significantly lower in SARS‐CoV‐2‐positive individuals (*p* = 0.016 at both species and ASV levels), supporting a potential association with infection status. This does not imply causality and should not be interpreted as a diagnostic marker. Additionally, *P. nanceiensis* abundance decreased with age, and because the SARS‐CoV‐2–positive group was younger than the negative group, this observation may partially reflect age‐related differences rather than infection‐specific effects. Regarding mucosa‐related symptoms, the Species and ASV *Veillonella parvula* was significantly depleted in symptomatic individuals (*p* = 0.001, *q* = 0.045) compared to SARS‐CoV‐2‐positive individuals without any mucosa‐related symptoms, with no other taxa reaching significance (Figure 2C). Although these associations did not remain significant after FDR correction (*q* > 0.05), consistency across taxonomic levels suggests they may represent exploratory but reproducible signals requiring independent validation. Among all taxa, *Prevotella nanceiensis* was the most consistently associated taxon with SARS‐CoV‐2 status, although this represents an observational association (Supporting Information S1: Table S3).

### 3.5 Temporal Dynamics: Oral Microbiome in Individuals Transitioning From SARS‐CoV‐2 Positive to Negative

We analyzed longitudinal data from 11 individuals who provided two oral samples, one during SARS‐CoV‐2 infection and other during the negative phase. The interval between visits ranged from 106 to 216 days, with a mean of 184.3 days (approximately 6.1 months).

**Figure 3:**
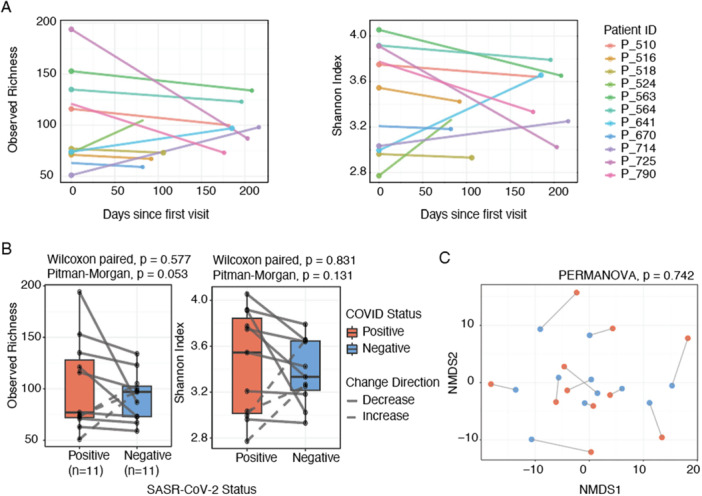
Temporal changes in oral alpha and beta diversity according to SARS‐CoV‐2 infection status among the sample individuals. (A) Shows individual trajectories of observed ASVs (richness) and Shannon diversity index over time since the first study visit, with lines colored by participant ID and point shapes indicating the COVID‐19 vaccination status. (B) Paired comparisons of alpha diversity between SARS‐CoV‐2‐positive and ‐negative oral samples from the same individuals, with solid gray lines indicating a decrease and dashed lines indicating an increase in diversity following viral clearance. (C) NMDS plot based on Aitchison dissimilarity. Boxplots represent the medians and interquartile ranges, with individual data points overlaid. P‐values correspond to paired Wilcoxon tests, Pitman‐Morgan for paired variance, and PERMANOVA.

There was a moderate, non‐significant positive correlation between the number of days between visits and an increase in oral microbial alpha diversity (Pearson's *r* = 0.44–0.46, *p *> 0.159), indicating that the time between visits had a minimal impact on microbial diversity (Figure 3A). This was calculated using paired Shannon index and observed richness values from the longitudinal samples of the same individuals. No significant differences in alpha diversity metrics were observed between SARS‐CoV‐2‐positive and ‐negative samples from the same individual (paired Wilcoxon test, all *p* > 0.577). Although most patients showed a decrease in alpha diversity after becoming SARS‐CoV‐2‐negative (*n* = 8 vs. *n* = 3), this trend was not statistically significant (Fisher's test, *p* > 0.050), and the small sample size limited robustness. However, the observed richness diversity variance marginally decreased with SARS‐CoV‐2 clearance (Pitman‐Morgan test, *p* = 0.053, Figure 3B). No significant differences in variance were observed for microbial alpha diversity (Shannon index, *p* = 0.131) or beta diversity (PERMANOVA, *p* = 0.742, *R*² = 2.5%; Figure 3C).

### 3.6 The Influence of Vaccination on the Oral Microbiome

**Figure 4:**
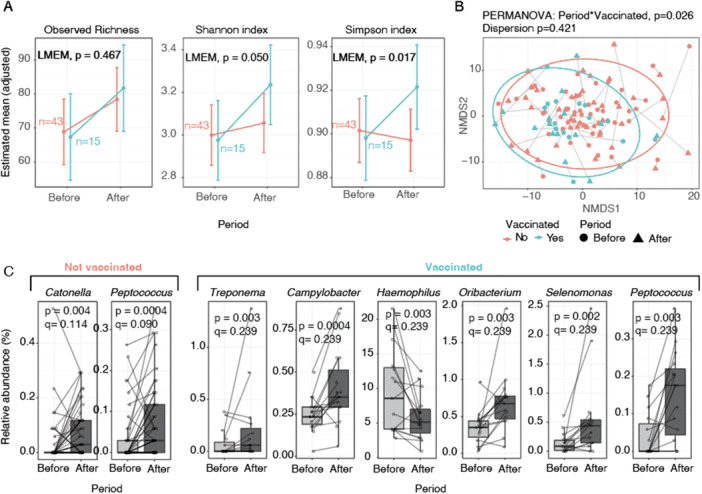
Temporal changes in the oral microbiome according to the vaccination status. (A) Oral alpha diversity indices (left: observed richness, middle: Shannon index, right: Simpson index). The points represent the estimated marginal means adjusted using linear mixed‐effects models (LMEM). with error bars indicating 95% confidence intervals. *p*‐values correspond to the interaction between period (Before vs After, mean 104 days) and vaccination status from LMEM. (B) Beta diversity (Aitchison distance) visualized with NMDS ordination. Ellipses represent 95% confidence intervals. P values were determined with PERMANOVA and PERMDISP. (C) Differentially abundant genera associated with vaccination status over time. Boxplots show the relative abundance (%) of selected taxa before and after the follow‐up period among non‐vaccinated and vaccinated individuals. Paired samples are connected with lines. *p*‐ and *q*‐values are derived from MaAslin2.

To assess whether COVID‐19 vaccination influences the oral microbiome, we compared samples collected before and after vaccination from confirmed SARS‐CoV‐2–negative individuals with no prior suspected infection. Baseline demographics, clinical factors, and lifestyle characteristics did not differ between those who remained unvaccinated (No→No) and those who became vaccinated (No→Yes) (Table 2). After adjusting for age, BMI, sex, and recent antibiotic use, we observed that individuals who had received a first vaccine dose (Pfizer‐BioNTech: *n* = 12, Moderna: *n* = 1, unspecified: *n* = 2; mean follow‐up < 104 days) showed significant increases in oral microbial diversity over time. Specifically, Shannon (*p* = 0.050) and Simpson (*p* = 0.017) diversity increased after vaccination, whereas observed richness did not show significant changes (Figure 4A). These results suggest that COVID‐19 vaccination may not expand the number of taxa present in the oral cavity but could influence their relative abundance. The increases in Shannon and Simpson diversity suggest a more even community structure after vaccination, potentially reflecting reduced dominance of a few taxa and greater balance across the oral microbiome.

**Table 2:** Participant characteristics at baseline stratified by incident COVID‐19 vaccination status (No→No vs No→Yes).

Variable	COVID‐19 vaccinated status (before‐> after)		
	No‐> No	No‐> Yes	*p* a
*n*	43	15	
Age (mean (SD))	42.44 (11.49)	40.33 (12.52)	0.552
Sex = Male (%)	5 (11.6)	2 (13.3)	1.000
BMI (mean (SD))	28.94 (5.91)	29.03 (10.81)	0.969
COVID19 vaccinated = Yes (%)	0 (0.0)	15 (100.0)	< 0.001*
Vaccine Brand = Phizer BioNTech (%)	0 (NaN)	12 (92.3)	NaN
Vaccine Doses = two doses (%)	0 (NaN)	13 (92.9)	NaN
Physical Activity Sports = Yes, > 0 days week (%)	2 (4.7)	3 (20.0)	0.197
Antibiotics last 2months = Yes (%)	5 (11.6)	2 (13.3)	1.000
Diabetes type 2 = Yes (%)	7 (16.3)	3 (20.0)	1.000
Nicotine cigarret curr = Yes (%)	1 (2.3)	2 (13.3)	0.327
Medicacal or recreatinal cannabis = Yes (%)	1 (2.3)	1 (6.7)	1.000
Hypertension = Yes (%)	11 (25.6)	6 (40.0)	0.467
Smoking curr vap cigar w and wo nicot = Yes (%)	2 (4.7)	2 (13.3)	0.582
Ever periodontal disease = Yes (%)	1 (2.3)	1 (6.7)	1.000
Rhinitis = Yes (%)	13 (30.2)	1 (6.7)	0.137
Bronchial asthma = Yes (%)	12 (27.9)	3 (20.0)	0.795
Ever cancer = Yes (%)	1 (2.3)	0 (0.0)	1.000

^a^ *p*‐values were generated from T‐test or Kruskal‐Wallis for continous variables or Chi‐squared or Fisher exact test for categorical variables.

* *p*‐values < 0.05.

Beta diversity analysis further supported this pattern, showing that vaccination status modified the temporal shift in community composition (PERMANOVA, *p* = 0.026), and no significant differences in dispersion were detected (*p* = 0.421, Figure 4B). Finally, differential abundance analysis revealed the specific taxa contributing to these changes. Among the vaccinated individuals, the relative abundances of *Treponema, Campylobacter, Oribacterium, Selenomonas*, and *Peptococcus* increased after vaccination, whereas *Haemophilus* decreased. In contrast, among the non‐vaccinated participants, changes were limited to an increase in *Catonella* and *Peptococcus* over time. The latter was also observed in vaccinated individuals, indicating that *Peptococcus* is unlikely to represent a vaccination‐specific response but instead reflects a temporal trend across the groups, which may reflect temporal or behavioral factors such as the more frequent use of masks by the population which was not fully assessed in the study (Figure 4C).

## 4 Discussion

In our Hispanic cohort sampled during the active phase of SARS‐CoV‐2 infection, we found no significant differences in alpha or beta diversity between infected and uninfected individuals. Instead, recent antibiotic exposure and age emerged as the strongest determinants of oral microbiome diversity. Beta diversity based on Aitchison distance showed only a weak, non‐significant trend toward separation by infection status. These results suggest that in community‐based, non‐hospitalized patients with ongoing SARS‐CoV‐2 infection, the oral microbiome remains relatively stable.

Our findings align with studies in asymptomatic or recovered populations, which also reported minimal effects of SARS‐CoV‐2 on oral microbial diversity. For example, (Jitvaropas et al. 2022) in Thailand found no differences in Shannon, Chao1, or Bray–Curtis diversity among asymptomatic cases and controls; similarly, in China reported no significant alpha or beta diversity differences in patients sampled after recovery (Wei et al. 2023). This convergence suggests that, outside of severe disease or hospitalization, SARS‐CoV‐2 alone may not consistently drive large‐scale shifts in oral microbial diversity.

By contrast, studies in hospitalized populations have reported more pronounced changes.(Gupta et al. 2017) and Bhanu et al. (2025)(Bhanu et al. 2025), both conducted in India, found significant reductions in Shannon, Simpson, and Chao1 indices, alongside clear compositional shifts by Bray–Curtis and Jaccard distances. Likewise, Brzychczy‐Sroka et al. (2023)(Brzychczy‐Sroka et al. 2023) in Poland observed significant reductions in alpha diversity and marked beta diversity differences in saliva and dental plaque from post‐hospitalized COVID‐19 patients. These discrepancies highlight the influence of clinical severity, treatment, and hospitalization on oral microbial outcomes, factors largely absent and besides the initial workplan in our outpatient cohort.

Methodologically, our use of Aitchison distance—well‐suited for compositional data—offers a more robust assessment of beta diversity compared with Bray–Curtis or Jaccard, the metrics most frequently used in prior studies. The consistency of our findings across models adjusted for antibiotics, age, and sex, combined with the absence of strong effects in community‐based cohorts from Thailand and China, strengthens the interpretation that oral microbiome diversity may be resilient to SARS‐CoV‐2 infection in the absence of hospitalization or severe disease.

Our analysis identified several oral taxa with nominal associations to SARS‐CoV‐2 infection. Specifically, *Prevotella jejuni, Prevotella salivae, Leptotrichia* sp., and Lachnospiraceae [G‐2] sp. were enriched in infected individuals, while *Rothia mucilaginosa* and *Prevotella nanceiensis* were depleted. After excluding antibiotic users, *P. nanceiensis* remained significantly reduced, highlighting its importance as a bacterial component of the oral cavity, and interpretation should be done with caution.

These results echo findings from other cross‐sectional studies. For instance, oral enrichment of *Prevotella* species during COVID‐19 has been reported in Italian cohorts, including periodontopathogenic *P. melaninogenica, P. jejuni, P. denticola*, and *P. oris* (Soffritti et al. 2021). Our detection of *P. jejuni* aligns with these results. Conversely, we found *P. nanceiensis* to be consistently depleted. Previous studies have reported reduced oral *Prevotella* genus in the Bangladeshi population (Rafiqul Islam and Foysal 2022), a finding that was not observed in our cohort. Importantly, while we identified *P. salivae* enrichment in infected individuals, a Mexican cohort reported higher *P. salivae* abundance specifically among hospitalized or deceased patients compared to asymptomatic or non‐hospitalized (L Serrato and Meza 2023), suggesting that although this species increases during infection in both populations, its association with disease severity may differ by context. We cannot discard the possibility that the observed taxonomic differences may be influenced by underlying oral health factors, including periodontal disease, caries activity, and oral hygiene status, which were not assessed in this study and were beyond its scope. Therefore, these associations should be interpreted with caution.

The depletion of *Rothia mucilaginosa* in our cohort also aligns with the Italian study, which reported reduced *Rothia* spp. in oral samples from infected patients (Soffritti et al. 2021), but contrasts with Chinese findings where oral *Rothia mucilaginosa* was enriched (Wu et al. 2021). Such inconsistencies may reflect geographic, host, or disease‐severity differences across populations.

Together, these comparisons highlight that while certain genera such as *Prevotella* and *Rothia* repeatedly emerge in association with COVID‐19, the direction and species‐level specificity of these shifts vary by study. The consistent depletion of *P. nanceiensis*, even after excluding antibiotic users, suggests an association with SARS‐CoV‐2 infection; however, given that infection is determined by diagnostic testing, this finding should be interpreted as an ecological association rather than a diagnostic marker.

### 4.1 Temporal Analysis

In a cohort of 11 individuals followed from SARS‐CoV‐2 infection to the clearance phase over approximately 6 months, we found no significant differences in alpha or beta diversity. However, there was a significant decline in alpha diversity variance after clearance, suggesting greater heterogeneity of oral alpha diversity during infection. These results align with Armstrong et al. (2023) (Armstrong et al. 2023), who reported stable salivary microbiota in mild or moderate infections, with diversity reductions only in severe cases that normalized over time (Armstrong et al. 2023. Similarly, Cui et al. (2022) showed gradual recovery of the oral microbiome over 1 year, though not complete, when compared to healthy controls (Cui et al. 2022). In contrast, Wu et al. (2021) and Paine et al. (2022) observed marked dysbiosis during acute infection that persisted for at least 30 days (Wu et al. 2020)(Paine et al. 2022). More recently, also found significantly reduced diversity one month after recovery, indicating lingering microbial alterations (Wei et al. 2023).

Compared to these studies, our data suggest that in primarily mild infections, the oral microbiome remains relatively stable, with interindividual alpha diversity heterogeneity during infection. This may reflect the resilience of the oral microbiota, with disease severity and follow‐up duration shaping whether transient or persistent changes are observed.

### 4.2 Influence of Vaccination

Our results indicate that COVID‐19 vaccination is associated with short‐term changes in the oral microbiome. However, these changes should be interpreted with caution, as they may reflect microbial resilience or immune‐mediated ecological restructuring rather than a direct clinical benefit. Within ~104 days after the first dose, Shannon and Simpson diversity increased, while richness remained unchanged. This suggests that vaccination does not expand the number of taxa but instead promotes a more even distribution across the community. Beta diversity analyses further showed that vaccination modified the temporal trajectory of community composition, indicating systematic shifts rather than random variation. Similar results in alpha and beta were obtained by other authors with similar ARNm‐type vaccination (Uehara et al. 2022).

At the taxonomic level, vaccinated individuals exhibited increases *in Treponema, Campylobacter, Oribacterium, Selenomonas*, and a decrease in *Haemophilus* as vaccination‐specific taxa change. Uehara's 2022 study found only *Bacteroides* to be reduced, and *Lachnoanaerobaculum, Moryella, Parvimonas*, and *Peptostreptococcus*, on the other hand, to be increased in vaccinated individuals (Uehara et al. 2022). The decline of *Haemophilus* may reflect ecological pressures or immune‐mediated effects. The biological meaning of these changes is not yet clear. Genera such as *Treponema* and *Selenomonas* include species linked to periodontal disease, but their increase occurred alongside higher diversity and evenness, conditions generally associated with resilience rather than dysbiosis (Bik et al. 2010; Wade 2021). Potential mechanisms include systemic immune activation after vaccination that extends to the oral mucosa, influencing local IgA responses, cytokine levels, or immune cell activity. Indirect changes, such as shifts in salivary physiology or host behavior post‐vaccination, may also contribute.

## 5 Conclusion

Our study demonstrates that in this Hispanic cohort with ongoing SARS‐CoV‐2 infection, the oral microbiome remains largely stable, with no significant differences in alpha or beta diversity between infected and uninfected individuals. Instead, age and recent antibiotic exposure were the main drivers of diversity although the SARS‐CoV‐2–positive group was mostly younger than the negative group which could pose a confoudng effect. These findings, consistent with reports from asymptomatic or recovered populations, suggest that SARS‐CoV‐2 infection alone does not induce substantial alterations in oral microbial diversity, except in the context of severe disease or hospitalization.

Although we identified several taxa nominally associated with infection—such as the enrichment of *Prevotella* species or *Leptotrichia* and the depletion of *Rothia*—the direction and magnitude of these shifts varied across studies. Notably, the consistent depletion of *P. nanceiensis* even after excluding antibiotic users highlights its association with SARS‐CoV‐2 infections. Causality cannot be inferred from this cross‐sectional analysis, and the observed patterns likely reflect shifts in oral microbial ecology during infection rather than a direct role of this taxon in disease.

Longitudinal analysis showed that the oral microbiome remained stable from the active to the post‐infection phase, with only a slight increase in alpha diversity heterogeneity during infection. This resilience aligns with prior work, which shows that pronounced dysbiosis tends to occur in severe cases and may normalize over time.

Finally, while SARS‐CoV‐2 infection did not substantially alter the oral microbiome, COVID‐19 vaccination was associated with transient increases in microbial evenness and shifts in beta diversity, reflecting a coordinated reorganization of the oral community structure.

Together, these findings underscore the relative stability and resilience of the oral microbiome in mild SARS‐CoV‐2 infection, highlight potential microbial association with the infection, and reveal that vaccination can transiently modulate microbial community structure. Future research should explore the mechanisms underlying these changes and their clinical significance, particularly in the context of disease severity, treatment, and long‐term oral and systemic health.

## Author Contributions

**Daniela Vargas‐Robles:** methodology, investigation, writing – review and editing. **Frances Vázquez:** methodology, data curation, writing – review and editing. **Anelisse Dominicci‐Maura, Jean L. Santos Agrait,**, and **Jaleniz Suarez‐Pérez:** methodology, investigation, writing – review and editing. **Carlos A. Sariol, Josefina Romaguera**, and **Carmen Zorrilla:** methodology, investigation, writing – review and editing, resources. **Filipa Godoy‐Vitorino:** methodology, investigation, formal analysis, validation, supervision, funding acquisition, project administration, writing – review and editing.

## Ethics Statement

Ethical approval for this study was obtained from the Institutional Review Board (IRB) of the University of Puerto Rico School of Medicine (protocol #B1760120), and authorization for the use of biological samples was granted by the Institutional Biosafety Committee (IBC protocol #94320). All study procedures followed established ethical guidelines and were conducted in accordance with the principles of informed consent. All participants provided written informed consent prior to their participation, and their anonymity and confidentiality were strictly maintained throughout the study.

## Conflicts of Interest

The authors declare no conflicts of interest.

## Supporting information


**Table S1:** Oral microbiome beta diversity analyses model results by SARS‐CoV‐2 status or any mucosa symptoms and the covariates. Results of PERMANOVA and beta dispersion models evaluating the association between SARS‐CoV‐2 status and other covariates with oral microbial community structure using Aitchison distances. The full model for PERMANOVA was: Beta diversity ~ SARS‐CoV‐2 status + antibiotic use +  sex + age. For beta disper obly the SARS‐CoV‐2 status or any mucosa symptoms was evaluated. Asterisks (*) indicate p < 0.050. The column ‘model number’ groups terms that belong to the same analysis. **Table S2:** Alpha diversity linear models of oral alpha diversity metrics. The full model included: Alpha Diversity ~ SARS‐CoV‐2 status + antibiotic use + BMI + sex + age. Covariates retained in each final model were selected using stepwise model selection. This table displays only the non‐intercept terms retained in the final models. The column ‘model number’ corresponds to each distinct final model, used to group terms belonging to the same analysis. Asterisks (*) indicate p < 0.050. **Table S3:** Nominally significant associations between microbial taxa and clinical or demographic variables across taxonomic levels. Taxa showing significance, at nominal level (p < 0.050; FDR‐adjusted q > 0.250) or complete significant (p < 0.050; FDR‐adjusted q < 0.250) across Maaslin2 models evaluating the association of oral microbiome composition with SARS‐CoV‐2 infection status (with and without recent antibiotic use), mucosa‐related symptoms, and covariates including age, and sex. Taxa are shown across multiple taxonomic levels (ASV to phylum), with each row indicating the variable of association, taxonomic classification, direction and strength of the effect (coefficient), and the corresponding p‐ and q‐values. The column “Analysis Source” indicates the origin of the model (e.g., “Without antibiotics” or “All samples”), while “Taxonomic Level” indicates the classification level of each feature. These results provide exploratory insight into potential associations that did not remain significant after multiple testing correction but may warrant further investigation.

**Figure S1:** Oral microbiome diversity according to SARS‐CoV‐2 infection status, mucosal symptoms, and antibiotic use. A–B. Alpha diversity indices (observed richness and Shannon index) among all participants (A) and only among individuals with no recent antibiotic intake (B), stratified by SARS‐CoV‐2 infection status. C–D. Alpha diversity indices (observed richness and Shannon index) among SARS‐CoV‐2–positive individuals (C) and only among those with no recent antibiotic intake (D), stratified by the presence of mucosal symptoms. P‐value from the alpha diversity analyses come from the adjusted linear model. A p‐value labeled “ns” indicates that the SARS‐CoV‐2 status variable was dropped from the best‐fitted model, meaning its influence is minimal. E–F. Beta diversity (Aitchison distance) visualized using non‐metric multidimensional scaling (NMDS) among those without recent antibiotic intake by SARS‐CoV‐2 infection status (E), or among SARS‐CoV‐2–positive individuals by mucosal symptoms, including all (F) or only individuals without recent antibiotic intake (G). Ellipses represent 95% confidence intervals. P‐values correspond to PERMANOVA and PERMDISP tests.

## Data Availability

The data that support the findings of this study are openly available in European Nucleotide Archive at https://www.ebi.ac.uk/ena/browser/view/PRJEB104336, reference number ERP185631. The raw sequences and their associated metadata have been deposited in ENA study number ERP18563 with accession number PRJEB104336.
