# Supplementary material for: Oral Microbiome Resilience During SARS‐CoV‐2 Infection and Diversity Shifts After COVID‐19 Vaccination in a Hispanic Population
Source: Microbiologyopen. 2026 May 6;15(3):e70310. doi: 10.1002/mbo3.70310 (PMC13149225; doi:10.1002/mbo3.70310)
Supplement: Supplementary file 2 — Figure S1: Oral microbiome diversity according to SARS‐CoV‐2 infection status, mucosal symptoms, and antibiotic use. A–B. Alpha diversity indices (observed richness and Shannon index) among all participants (A) and only among individuals with no recent antibiotic intake (B), stratified by SARS‐CoV‐2 infection status. C–D. Alpha diversity indices (observed richness and Shannon index) among SARS‐CoV‐2–positive individuals (C) and only among those with no recent antibiotic intake (D), stratified by the presence of mucosal symptoms. P‐value from the alpha diversity analyses come from the adjusted linear model. A p‐value labeled “ns” indicates that the SARS‐CoV‐2 status variable was dropped from the best‐fitted model, meaning its influence is minimal. E–F. Beta diversity (Aitchison distance) visualized using non‐metric multidimensional scaling (NMDS) among those without recent antibiotic intake by SARS‐CoV‐2 infection status (E), or among SARS‐CoV‐2–positive individuals by mucosal symptoms, including all (F) or only individuals without recent antibiotic intake (G). Ellipses represent 95% confidence intervals. P‐values correspond to PERMANOVA and PERMDISP tests. [file MBO3-15-e70310-s002.docx]

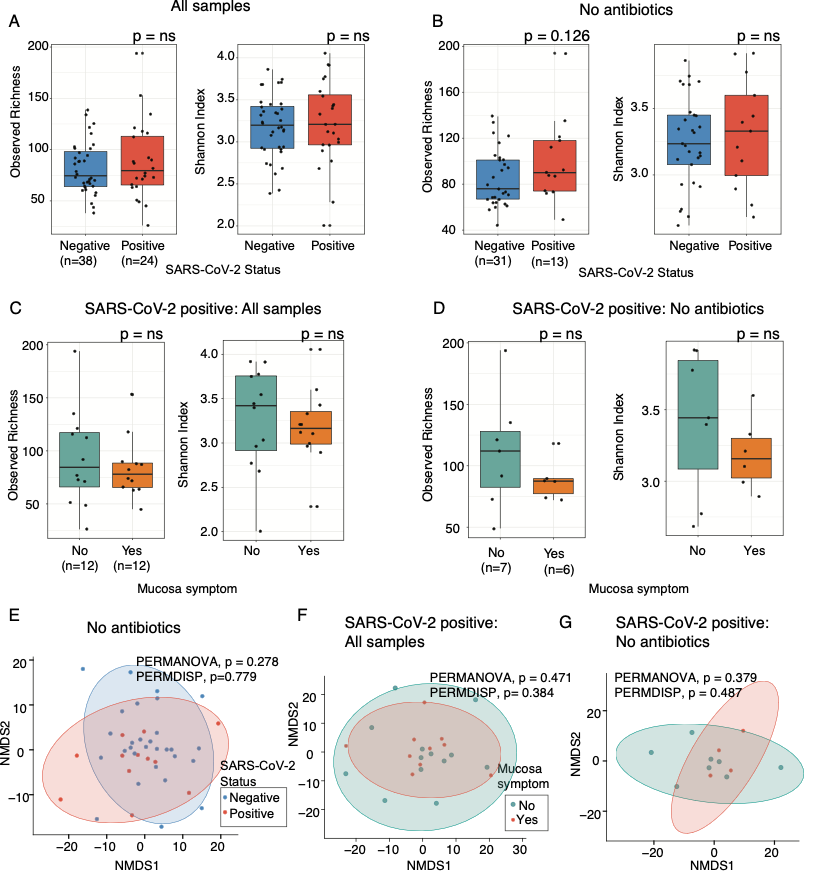


**Figure S1. Oral microbiome diversity according to SARS-CoV-2 infection status, mucosal symptoms, and antibiotic use.** **A–B.** Alpha diversity indices (observed richness and Shannon index) among all participants **(A)** and only among individuals with no recent antibiotic intake (**B**), stratified by SARS-CoV-2 infection status. **C–D.** Alpha diversity indices (observed richness and Shannon index) among SARS-CoV-2–positive individuals (**C**) and only among those with no recent antibiotic intake **(D)**, stratified by the presence of mucosal symptoms. P-value from the alpha diversity analyses come from the adjusted linear model. A p-value labeled “ns” indicates that the SARS-CoV-2 status variable was dropped from the best-fitted model, meaning its influence is minimal. **E-F.** Beta diversity (Aitchison distance) visualized using non-metric multidimensional scaling (NMDS) among those without recent antibiotic intake by SARS-CoV-2 infection status (**E**), or among SARS-CoV-2–positive individuals by mucosal symptoms, including all (F) or only individuals without recent antibiotic intake (**G**). Ellipses represent 95% confidence intervals. P-values correspond to PERMANOVA and PERMDISP tests.
